# Yes or no? A study of ErrPs in the “guess what I am thinking” paradigm with stimuli of different visual content

**DOI:** 10.3389/fpsyg.2024.1394496

**Published:** 2024-07-24

**Authors:** Artemiy Berkmush-Antipova, Nikolay Syrov, Lev Yakovlev, Andrei Miroshnikov, Frol Golovanov, Natalia Shusharina, Alexander Kaplan

**Affiliations:** ^1^Baltic Center for Neurotechnology and Artificial Intelligence, Immanuel Kant Baltic Federal University, Kaliningrad, Russia; ^2^Laboratory for Neurophysiology and Neuro-Computer Interfaces, Department of Human and Animal Physiology, Faculty of Biology, Lomonosov Moscow State University, Moscow, Russia

**Keywords:** ERN, error-related potentials, ErrPs, BCI, neurofeedback, brain-AI interaction

## Abstract

Error-related potentials (ErrPs) have attracted attention in part because of their practical potential for building brain-computer interface (BCI) paradigms. BCIs, facilitating direct communication between the brain and machines, hold great promise for brain-AI interaction. Therefore, a comprehensive understanding of ErrPs is crucial to ensure reliable BCI outcomes. In this study, we investigated ErrPs in the context of the “*guess what I am thinking*” paradigm. 23 healthy participants were instructed to imagine an object from a predetermined set, while an algorithm randomly selected another object that was either the same as or different from the imagined object. We recorded and analyzed the participants’ EEG activity to capture their mental responses to the algorithm’s “predictions”. The study identified components distinguishing correct from incorrect responses. It discusses their nature and how they differ from ErrPs extensively studied in other BCI paradigms. We observed pronounced variations in the shape of ErrPs across different stimulus sets, underscoring the significant influence of visual stimulus appearance on ErrP peaks. These findings have implications for designing effective BCI systems, especially considering the less conventional BCI paradigm employed. They emphasize the necessity of accounting for stimulus factors in BCI development.

## 1 Introduction

Non-invasive brain-computer interfaces (BCIs) translate user intentions, detected as changes in brain activity (e.g., electroencephalography (EEG) signal), directly into control signals for external devices without requiring muscle activity (Wolpaw et al., 2002; Abiri et al., 2019; Wolpaw et al., 2020). In this approach, the user’s mental effort generates specific patterns in brain signals that are recorded, classified, and translated into actionable commands (Wolpaw et al., 2020). Despite significant advances in BCIs, only a few mental strategies have been demonstrated to produce detectable brain signal perturbations for effective control (Abiri et al., 2019).

In addition, BCIs face certain limitations that lead to errors in recognizing user intentions. These errors can be caused by external factors, such as noise signals, insufficient spatial resolution of the EEG, and suboptimal feature extraction and classification algorithms. Internal factors, such as low level of attention and difficulties in performing mental tasks, also contribute to these errors (Moore, 2003; Zhang et al., 2021). To address this challenge, researchers have incorporated verification procedures that monitor the user’s mental responses to BCI output. This includes the detection of specific EEG responses, namely error-related potentials (ErrPs) or feedback-related potentials, which are neural signatures indicating mental agreement/disagreement with the presented BCI output (Chavarriaga et al., 2014). Detection of error-related activity makes it possible to correct the BCI feedback, thereby improving BCI performance (Chavarriaga et al., 2014; Yasemin et al., 2023).

Although ErrPs are useful for improving BCI performance, validation mechanisms have their own limitations. First, the reliance on decoding mental responses to validate BCI feedback significantly increases the cognitive load, resulting in a more complicated interaction paradigm (Plass-Oude Bos et al., 2011; Lotte, 2012; Ke et al., 2015). Users must shift their focus between different tasks, such as visualizing movements and the task of cognitive confirmation of BCI output. In addition, the current BCI paradigm often exhibits a mismatch between the intended mental commands and the actual output of the BCI system (Wang et al., 2022). For example, scenarios where users control robot movements with a P300-based BCI by mentally counting visual stimuli flashes (Gillini et al., 2022) or by imagining tongue movements (Guo et al., 2020) highlight this discrepancy. Such discrepancies demonstrate the lack of naturalness in brain-machine interaction.

To address these limitations, a new approach similar to reinforcement learning has been proposed in recent studies (Ferrez and Millán, 2005; Choi and Kim, 2019; Batzianoulis et al., 2021). In this paradigm, a user observes a robot’s behavior and controls it through mental approval or disapproval, which is detected by analyzing ErrPs. Consequently, this real-time feedback iteratively improves the robot’s performance. Unlike classical approaches that measure brain signals directly related to mental intent, in this new scenario, the computer presents various output options (e.g., different robot motion trajectories) and analyzes the neural responses to verify the validity of these potential outcomes (Batzianoulis et al., 2021). The validation process aims to determine whether the user agrees or disagrees with a suggested behavior of the machine or AI agent. If there is a conflict, the behavior is adjusted and the new option is tested. This approach is believed to provide a more natural user experience, similar to a cooperative interaction, through the principle of suggestion-confirmation/rejection (Ferrez and Millán, 2008; Omedes et al., 2018; Choi and Kim, 2019).

Building on this, we introduce a paradigm similar to the game “*guess what I am thinking*.” In this paradigm, the user imagines an object or a scene, and a second player (here represented by a machine algorithm) tries to guess the object by making assumptions. ErrPs are used as signatures of mental cues to confirm or reject the algorithm’s hypotheses. It is known that error-related EEG perturbations demonstrate high variability, which varies not only between individuals but also depending on the experimental paradigm (Iturrate et al., 2014; Abu-Alqumsan et al., 2017), error frequency and BCI outcome significance (Holroyd and Coles, 2002; LoTemplio et al., 2023). The emotionality of the presenting stimuli affects the shape of ErrPs (Larson et al., 2006). Furthermore, it is well studied that cortical responses are influenced by context within which stimuli are presented (Wlotko and Federmeier, 2012), the presence of faces and emotions they express (Bötzel and Grüsser, 1989), and the aesthetic qualities of stimuli (de Tommaso et al., 2008). In light of the above considerations, we propose that the appearance of the stimulus may influence the responses evoked by correct and incorrect feedback in BCI. In this study, we aimed to analyze the spatiotemporal characteristics of ErrPs in this proposed collaborative paradigm.

In this study, we asked participants to play a game with a computer in which they imagined a particular picture from a set, and the machine, as explained to the participants, would guess the imagined picture by presenting it on a screen. Observing the machine’s response was expected to elicit specific EEG signatures of mental agreement or disagreement in the participants. Given the wide range of scenes and objects that can be imagined, we used four different visual stimulus sets with different appearance and semantics (*objects*, *animals*, *emojis*, and *numbers*). Our primary goal was to investigate the consistency of the shape of the ErrPs across these different visual stimuli when presented as a machine response in a BCI loop. We hypothesized that the shape of ErrPs would be affected by the distinct visual semantic content of the stimuli, rather than remaining invariant, despite evidence suggesting that ErrPs are relatively conservative in shape for different feedback types (Iwane et al., 2022). We expected ErrPs in the studied paradigm would not only be shaped by components related to the processing of observed errors (Ferrez and Millán, 2005; Xavier Fidêncio et al., 2022; Syrov et al., 2023) but also by the processing of the content of the visual stimuli themselves. Also, our paradigm presents not a homogeneous N-back (namely *0-back*) type problem, but a constantly updating one, which is more similar to the actual interaction with BCI.

The results of this study may serve as a foundation for further advancement in the development of collaborative BCIs and significantly enhance our understanding of how feedback content affects ErrP shapes. It is crucial for researchers to recognize that, in addition to categorizing feedback as correct or incorrect, the content of stimuli may also influence ErrP shapes. Consequently, ErrP-based feedback validation procedures may be enhanced by considering the content and semantics of the stimuli.

## 2 Materials and methods

### 2.1 Participants and ethical considerations

A total of 23 healthy volunteers (11 males, mean age = 23 ± 4.5 years, right-handed) were recruited for this study. Eligibility criteria included the absence of self-reported neurological disorders, injuries, or other impairments of the nervous system. Detailed information about the research protocol was provided to all participants, and informed consent was obtained prior to participation in the study. The experimental design and procedures were reviewed and approved by the Ethics Committee of the Immanuel Kant Baltic Federal University.

### 2.2 Experimental design

To investigate ErrPs in the collaborative paradigm, the fake BCI paradigm was chosen, as its validity for studying human-machine interaction was detailed by Lynn et al. (2010), Évain et al. (2016), Lopez-Sola et al. (2021). Following the approach of Lynn et al. (2010), participants underwent specific procedures to enhance their belief in working with a genuine BCI. All participants were novices to BCI. Prior to experimental sessions, during EEG cap preparation, participants dedicated 40 min to understanding the principles of BCI technology, capable of decoding mental states through brain signal processing. Participants were also informed they would use similar technology during the experiments, fostering confidence in working with a BCI predicting their imagined images.

**Figure 1:**
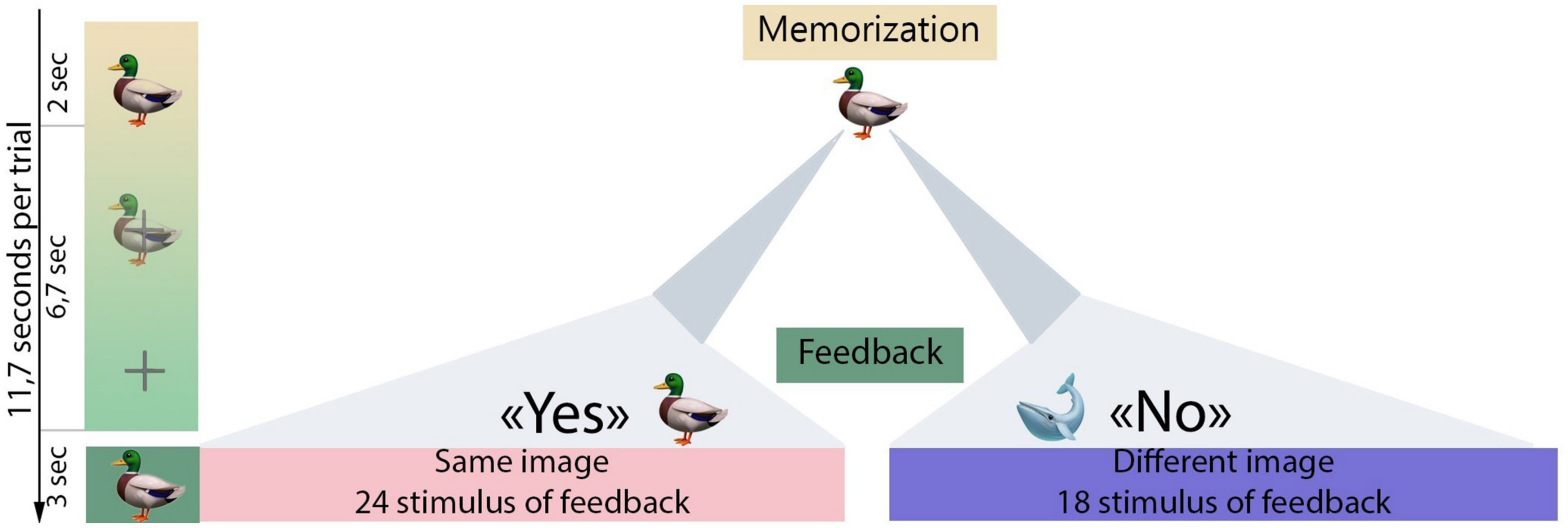
Experimental design. The gradient green bar illustrates the stimulus presentation sequence used in the experimental session. Initially, the first stimulus (e.g. a duck) was displayed for 2 s (memorization period). Then, over the next 6.7 s, the first stimuli gradually dissolved into a fixation cross (imagination period). After a 6.7 s interval from the onset of the first stimulus, the feedback was presented for 3 s. If the feedback image matched the initial stimulus (indicating a *Yes* response), participants were expected to mentally agree with the feedback presented. Conversely, if there was no match (indicating a *No* response), participants were instructed to mentally disagree with the feedback. After the feedback presentation, participants were given control to continue the experimental procedure by pressing a button on the keyboard (*self-paced* design).

There were four consecutive runs for each participant, with 42 trials in each run. Each trial in the experimental session comprised three stages: (1) Memorization period, the first presentation of a particular visual stimulus which participants were instructed to memorize; (2) Imagination period during which participants were encouraged to mentally reconstruct the image from the stimulus; (3) Feedback, the presentation of a visual stimulus that, as it has been instructed to the participants, is the result of a computer prediction of what picture the participants have imagined. The memorization period took 2 s, after which the stimulus image gradually disappeared for 2.2 s until it disappeared completely and the fixation cross appeared in its place. This lasted for 4.5 s (totaling the 6.7 s imagination period) until feedback. The feedback stimulus was presented for 3 s, then participants could advance to the next trial by pressing space on a keyboard. This was a *self-paced* approach that allowed participants to control the pace of the study by pressing a button to advance to the next trial start. If the button was not pressed, the next trial started automatically 15 s after the end of the previous trial. Feedback stimuli could be identical to the initially presented stimuli or different. During feedback, participants assessed whether it matched the initial stimulus, mentally agreeing or disagreeing with the computer’s “prediction”. The number of correct (mental response *Yes*) and incorrect (mental response *No*) feedback events was predetermined and amounted to 24 and 18, respectively, resulting in an accuracy of 57.1% (total number of trials *n* = 42). The order of trials with correct and incorrect feedback was randomized for each run. The fake-BCI paradigm aimed to boost participant’s motivation. A visual representation of the experimental session design is presented in Figure 1.

#### 2.2.1 Stimulus sets

The study used four different stimulus sets, each containing different types of visual stimuli. Each stimulus set was designed to represent different semantic domains: set#1 contained images of various everyday objects and tools (referred to as *objects*), set #2 consisted of animal emojis (referred to as *animals*), set#3 contained numbers (referred to as *numbers*), and set#4 contained emojis depicting different facial expressions (referred to as *emojis*). Each set contained seven unique images with a black background. Further details on the composition of the stimulus sets can be found in Appendix 1.

### 2.3 EEG data acquisition

EEG data were recorded using a BrainVision actiCHamp+ amplifier (Brain Products GmbH, Germany) with 64 active channels. The sampling rate was set to 1,000 Hz. The placement of the active Ag/AgCl sensors followed the international 10/10 electrodes placement system. The TP10 channel position was used for the reference. To ensure optimal signal quality, the impedance between the electrodes and the skin was kept below 10 kΩ. During recording, a photo sensor was used to accurately mark the onset of stimulus presentation. A custom written Python script was used for stimulus presentation.

### 2.4 Data preprocessing and analysis

The raw EEG signal was downsampled to 500 Hz, then band-pass filtered in the 1–15 Hz range using a causal finite impulse response (FIR) filter. Interpolation of noisy channels was performed using the spherical spline method (Perrin et al., 1989). Independent Component Analysis (*fastICA* method) was applied to remove oculographic artifacts: the components highly correlated with Fp1 and Fp2 signals were excluded from the recording. The signal was then re-referenced using the common average reference (CAR) (McFarland et al., 1997). The preprocessed signal was then segmented into 900 ms epochs based on the photo sensor triggers. Each epoch started 100 ms before and ended 800 ms after the onset of the feedback stimulus. A baseline correction was applied using the time interval (−100; 0 ms).

In order to obtain ERPs related to mental (dis-)agreement, the epochs for each stimulus set were averaged separately according to the correctness of the feedback stimulus. In accordance with previous studies on ErrPs, which have localized the signal sources of these potentials (Falkenstein et al., 2000), we averaged epochs across the fronto-central channel group (*F1, Fz, F2, FC1, FCz, FC2, C1, Cz, C2*). This also allowed us to improve the signal-to-noise ratio.

Peak amplitudes were calculated using the following criteria: each peak was defined as the extreme value within a given latency range, and the mean peak value was taken within ±20 time samples around this value. Since we detected three pronounced peaks in *No* responses – an early positive peak and two peaks related to error-related negativity (ERN) and error-related positivity (Pe) – we used the following latency intervals for peak amplitude estimation: the maximal value for the early positive peak was found in the 140–260 ms range, the minimal value for the ERN peak was found in the 200–340 ms range, and the maximal value for the Pe peak was found in the 340–540 ms range, all in accordance with the stimulus onset. To obtain the corresponding values for *Yes* responses, the same latencies estimated from *No* responses were taken.

### 2.5 Statistical analysis

Before assessing the effects of feedback correctness and stimulus set on distinct components, we analyzed the distribution of peak amplitude values for all three components. Since the data did not follow a normal distribution according to the Shapiro–Wilk tests, we used the Friedman test to identify the influence of the *stimulus set* factor on the amplitude of all identified ERP components in *Yes* and *No* responses.

Subsequently, peak amplitudes for all three components were compared between *Yes-* and *No*-trials for each stimulus set, separately, utilizing the Wilcoxon signed-rank test. The Bonferroni correction was applied by multiplying the *p*-values by the number of paired comparisons performed.

To comprehensively explore the spatio-temporal characteristics of components in feedback-related responses and determine whether their appearance was significantly related to *Yes/No* categorization rather than the processing of stimulus content, we subtracted ERPs elicited by stimuli from the memorization period from the fake-feedback evoked activity. A spatio-temporal cluster-based permutation test with 10,000 permutations (Maris and Oostenveld, 2007) was employed to assess the result of the subtraction with zero. This statistical analysis allowed us to identify clusters related to components specifically associated with participants’ mental agreement with computer guesses.

For the EEG data processing and statistical analysis, we used self-written Python (v3.10) scripts utilizing the libraries MNE v1.5.0 (Gramfort et al., 2013), NumPy v1.26.0 (Harris et al., 2020), and SciPy v1.11.0. (Virtanen et al., 2020).

## 3 Results

**Figure 2:**
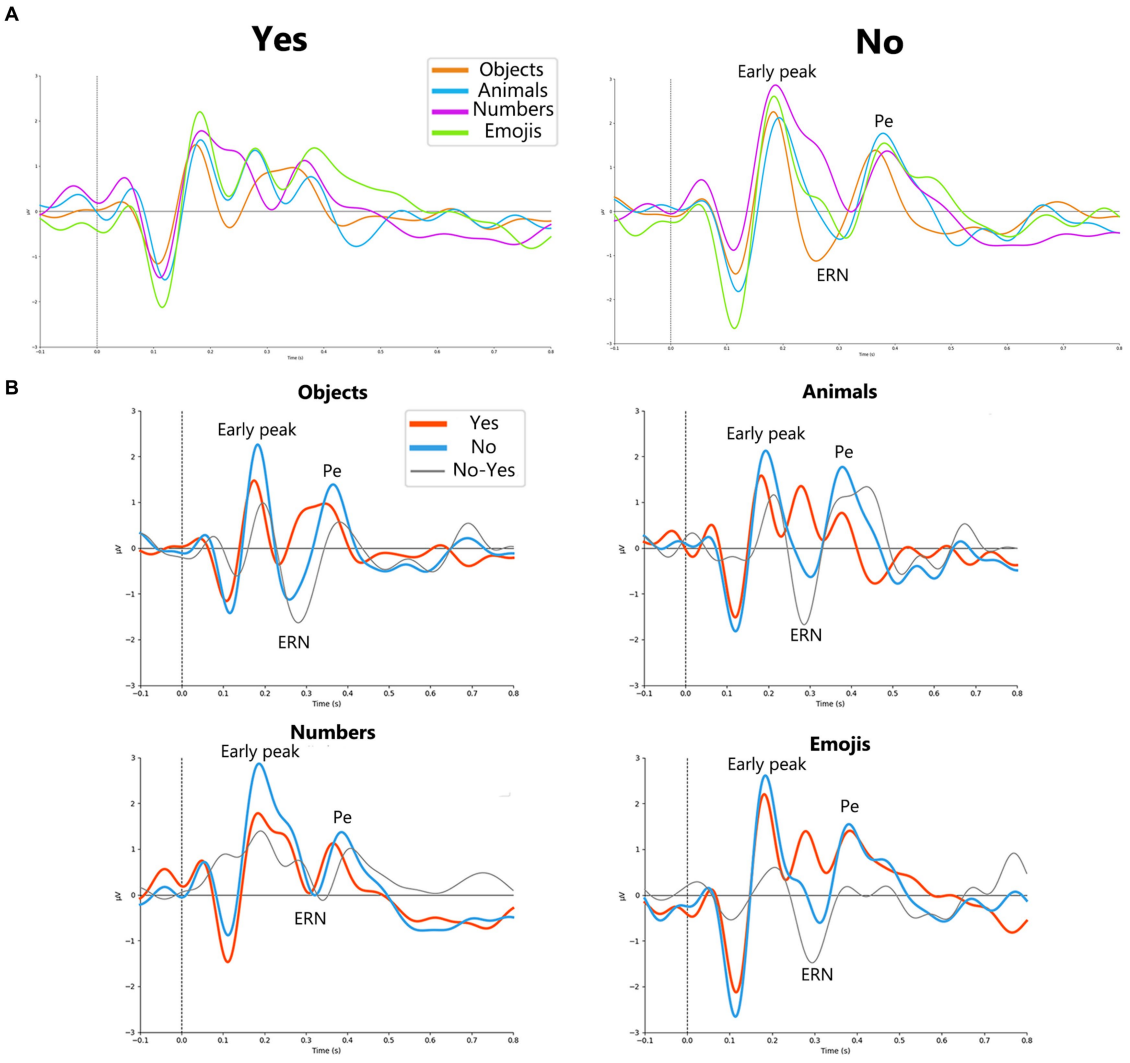
**(A)** *Yes* (on the left) and *No* (on the right) responses for each stimuli type *objects*, *animals*, *numbers* and *emojis* (orange, blue, purple and green lines, respectively). For *No* responses, early peak, ERN and Pe peaks are allocated in a group of channels (*F1, Fz, F2, FC1, FCz, FC2, C1, Cz, C2*) electrodes. **(B)** Grand average (N = 23) ErrPs for every stimulus set in a group of channels (*F1, Fz, F2, FC1, FCz, FC2, C1, Cz, C2*). Orange solid line represents *Yes* responses, blue solid line – *No* responses, black solid line – difference curve.

Analysis of ERPs related to *Yes* and *No* responses revealed distinct peaks between the two reactions, including the early positive peak, the negative ERN peak, and the late positive Pe peak. Figure 2A shows the curves for both *Yes* and *No* for all stimulus sets.

The results of the Friedman tests are presented in Table 1. The results demonstrated a significant effect of the *stimulus set* factor on the *amplitude* of the ERN component for *No* responses and on the *amplitude* of the Pe component for *Yes* responses.

**Table 1:** Friedman tests results for the *Yes* and *No* responses between different stimuli for each ERP component (early peak, ERN, Pe).

	Early peak		ERN		Pe	
	*F*	*p*	*F*	*p*	*F*	*p*
No	0.11247	0.27978	***0.18000***	***0.04300***	0.09400	0.43000
Yes	0.02493	2.10182	0.01607	2.46485	***0.20111***	***0.02840***

*p*-values were corrected using the Bonferroni method. Significant comparisons are highlighted in bold italics.

Further, we compared the amplitudes of the revealed components between *Yes* and *No* responses separately for each set of stimuli.

**Figure 3:**
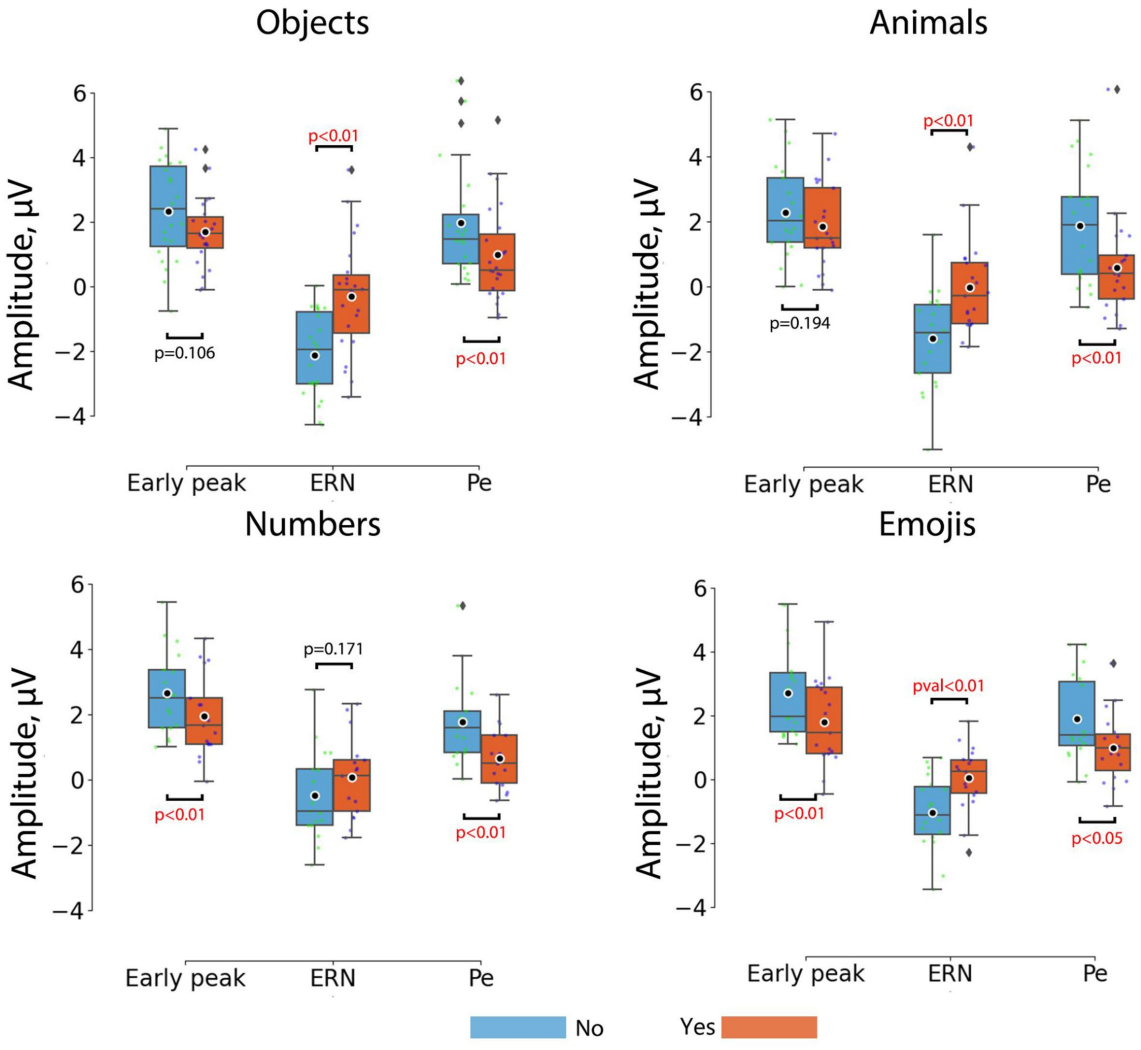
Comparisons between the peak amplitudes of ERP components for *No* (blue) and *Yes* (orange) responses across different stimulus sets. Horizontal lines within the boxes correspond to the median values, boxes – to the interquartile range and [Q1-1.5*IQR; Q3 + 1.5*IQR] range is shown by whiskers. Black circles represent mean values for the group. Colored dots represent individual values. Corrected *p*-values are shown for the Wilcoxon signed-rank test with Bonferroni correction.

Figure 2B shows the ERP curves for *Yes* and *No* responses for different stimulus sets, averaged over the group of channels. Detailed results for paired comparisons of ERP peaks for *Yes* and *No* responses are shown in Figure 3.

For the *objects* the most prominent ErrP peak was the ERN peak (*W* = 14; *p* = 0.00008), which appeared on average at around 266 ms, and the Pe peak (*W* = 18; *p* = 0.00018), which appeared at around 386 ms. For the *animals*, the most prominent ErrP peaks were also the ERN peak (*W =* 12; *p =* 0.0002) and the Pe peak (*W =* 29; *p =* 0.0048), appearing at approximately 304 ms and 402 ms, respectively. For the *numbers*, the most prominent peaks were the early peak (*W =* 10; *p =* 0.00197) and the Pe peak (*W =* 4; *p =* 0.00032), appearing at approximately 192 ms and 406 ms, respectively. For the *emojis* all peaks were prominent, although the early positive (*W =* 24; *p =* 0.00851) and ERN peaks (*W =* 12; *p =* 0.0008) were more pronounced compared to the Pe peak (*W =* 32; *p =* 0.02836), which appeared at approximately 178 ms (early positive peak), 308 ms (ERN) and 428 ms (Pe).

Thus, the early positive and ERN peaks were not robust to differences in stimulus material in the paradigm used, although the ERN occurred for 3 out of 4 stimulus sets (*objects*, *animals*, *emojis*) and the early positive peak occurred for the *numbers* and *emojis*. Although the significance levels for the Pe varied depending on the type of visual stimuli, it was the most stable component of the ErrP (Figure 2B).

**Figure 4:**
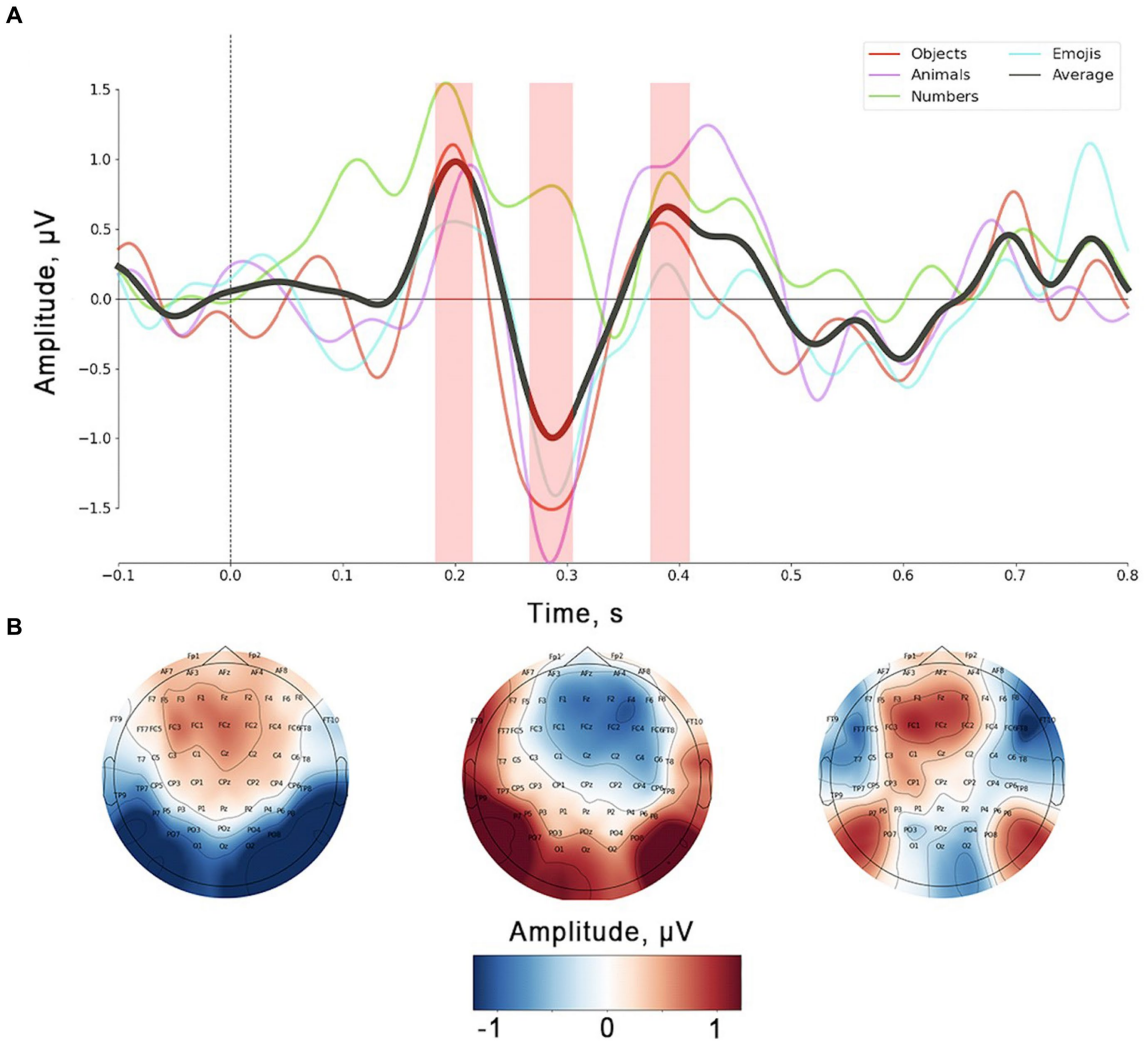
**(A)** Grand average (*N* = 23) of ErrPs (data presented for *FCz* channel). Color lines represent single stimulus sets, the black solid line corresponds to average value across all stimulus sets. Shaded area corresponds to the peaks’ time ranges used for statistical analysis and topographic mapping. **(B)** Topographic localization for three peaks observed on the ErrP curve.

Figure 4 depicts the distinctions between *No*-ERPs and *Yes*-ERPs for each stimulus set, including the average difference at electrode *FCz* (Figure 4A). The topographies presented illustrate the signal distribution for each identified peak (Figure 4B). The early positive peak was most prominent in the fronto-central channels (Figure 4B, leftmost topographic projection), with an average latency of 185 ms from stimulus onset. The amplitude and latency of the early positive peak vary slightly depending on the stimulus set, with the largest differences observed for the *numbers*, as noted during the analysis of ErrPs in individual sets. The ERN peak showed a fronto-central spatial distribution with a slight right lateralization (Figure 4B, topographic projection in the middle). This peak was usually observed approximately 300 ms after stimulus onset. The shape of the ERN peak remained consistent across the three stimulus sets but tended to show a delayed onset and reduced amplitude for the *numbers*. The spatial distribution of the Pe (Figure 4B, rightmost topographic projection) demonstrates a tendency to appear in fronto-central channels, similar to other components. On average, this peak was observed approximately 400 ms after the feedback presentation.

**Figure 5:**
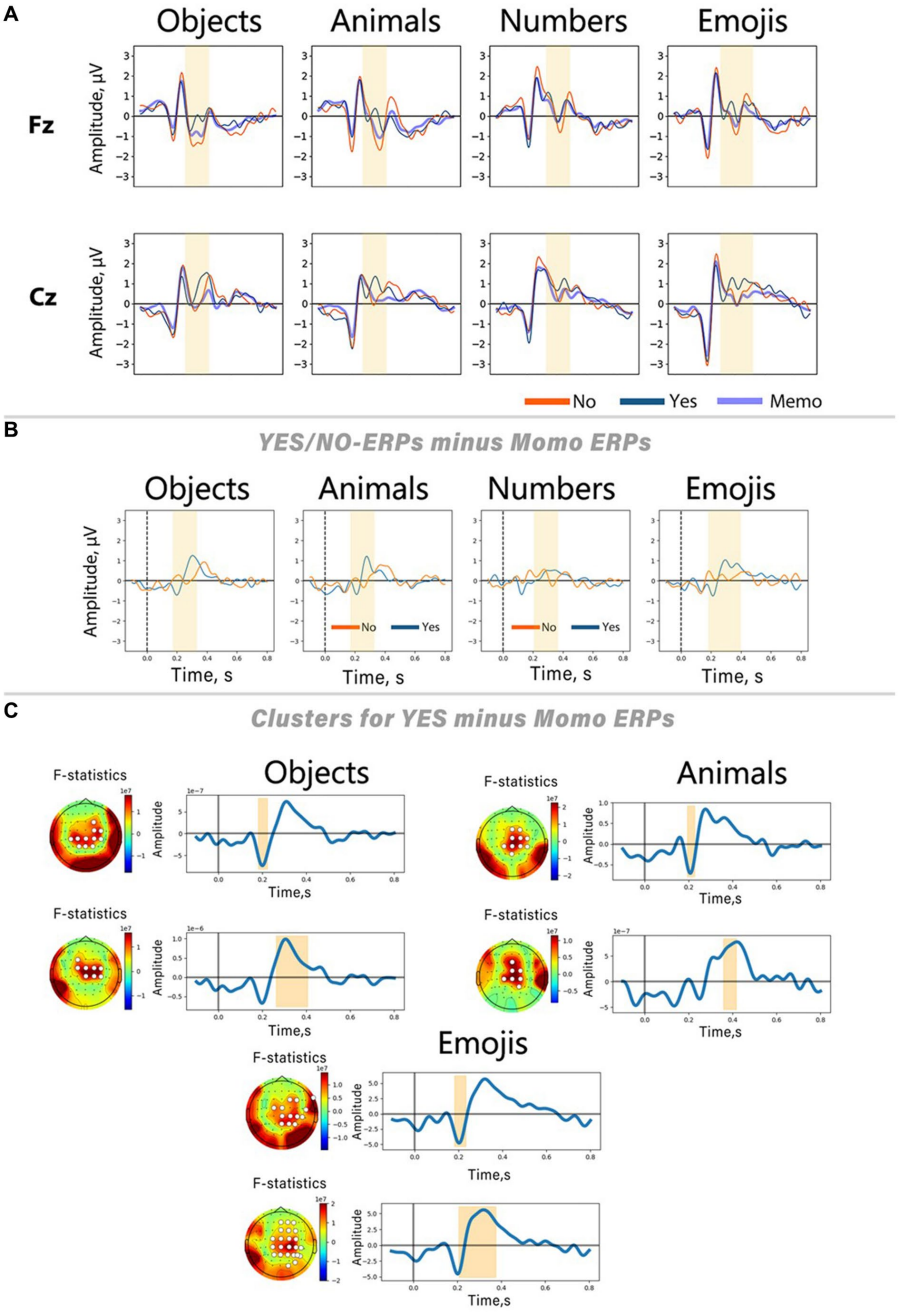
Differences between Yes/No-related ERPs and ERPs evoked by stimuli presented in memorization trials. **(A)** ERP curves from Fz and Cz channels for all stimulus sets. **(B)** Result of subtracting memorization-related ERPs (Memo ERPs) from Yes/No-related ERPs. ERPs from the Cz channel. Blue lines represent ERPs elicited during memorization trials. **(C)** Results of the spatio-temporal permutation test for three out of four sets (*objects, animals, emojis*). On the left side of each panel, the topographic distribution of the F-statistic averaged over the time interval associated with the significant cluster; white circles indicate channels belonging to the cluster. On the right side, curves obtained by subtracting the memorization-related ERP from the Yes responses, averaged over all channels in the cluster. The shaded areas indicate the time interval during which significant differences were found.

In Figure 5A, *Yes* and *No* responses are juxtaposed with ErrPs triggered by simple picture presentation during the memorization period. The figure exhibits a notable overlap between *No*-ERPs and ERPs associated with stimuli from memorization trials. To scrutinize the impact of components related to mere image content processing on ERPs associated with *Yes*/*No* categorization during computer response perception, we conducted a subtraction of memorization-related ERPs from those related to *Yes* and *No* responses (Figure 5B). This method allowed us to pinpoint peaks specifically linked to error and correct outcomes processing by eliminating potentially contaminating components. For statistical validation of the resulting peaks on the subtracted curves, a non-parametric spatio-temporal permutation test was employed.

The results of the Friedman tests revealed an effect of the *stimulus set* factor on the ERN *peak amplitude* in *No* responses, and late positive component *peak amplitude* in *Yes* responses. Its instability was also confirmed by analyzing the results of Wilcoxon signed-rank tests for each individual stimulus set. Our findings indicate that only *Yes* responses exhibited significant differences from the ERPs of memorization trials. Conversely, *No* responses showed no significant clusters according to permutation testing. Spatio-temporal clusters derived for the *Yes* responses reveal two peaks: a slight negative deflection with a latency of approximately 200 ms after the stimulus onset, succeeded by a positive peak within the time interval corresponding to the ERN deflection on the ErrP curve (~250–400 ms after the stimulus onset). These peaks exhibit similar shapes and spatial characteristics across all stimulus sets, underscoring their specificity for processing correct feedback. Interestingly, no clusters were found for the *numbers*, consistent with the findings that this stimulus set did not show significant differences between *Yes* and *No* responses within the time window related to the ERN peak.

## 4 Discussion

While ongoing endeavors to leverage ErrPs for BCI control have predominantly concentrated on enhancing spelling technologies relying on P300 and other evoked potentials (Dal Seno et al., 2010; Cruz et al., 2017), an equally noteworthy alternative is to consider ErrPs as the primary control EEG signal (Batzianoulis et al., 2021). Through establishing a framework of continuous interaction between the user and the “AI agent” via a *suggestion-confirmation/rejection* dialogue, our fake-BCI paradigm strived to recreate a real BCI user experience. Participants were tasked with memorizing a visual stimulus and providing a mental *Yes* or *No* response based on whether the computer-presented feedback stimulus matched the memorized one. In line with existing literature, our study confirmed that incorrect stimuli induced error-related ERPs characterized by a negative peak around 300 ms, followed by a subsequent positive deflection (Falkenstein et al., 2000; Glazer et al., 2018).

An early positive peak with a latency of approximately 150–250 ms was evident across all stimulus sets in both *Yes* and *No* responses. However, the *numbers* and *emojis* stimulus sets showed significant differences between *Yes* and *No* responses in this component. This may be due to differences in detail and color. We interpret numbers and emojis as simple, mostly monochromatic stimuli with few details, and according to a previous study by Michie et al. (1999), less detailed stimuli are easier to process and integrate. Our results suggest that visual attention systems are more responsive to simple stimuli in the BCI framework. Consequently, the task of comparing two visually simple stimuli can elicit signatures of image comparison even in the early stages of stimuli integration.

The negative peak observed in the *No* responses around 300 ms after the feedback stimulus, classified as the ERN, mainly manifested in the fronto-central group of channels. The spatial distribution and latency of this peak align with findings from previous neuroimaging studies focused on error processing (Gehring et al., 1993; Gehring and Fencsik, 2001), which associated this activity with detecting discrepancies between expected and actual outcomes (Bellebaum and Daum, 2008; Glazer et al., 2018). In our study, the amplitude of this negative deflection was greater in *No*-trials, whereas *Yes*-trials exhibited a positive deflection within the same latency range. As a result, the difference curve obtained by subtracting *Yes*-trials from *No*-trials revealed a distinct negative peak in the ErrP curve (Figure 3). This ErrP profile has been observed in paradigms involving both user behavioral errors and BCI errors (Ferrez and Millán, 2005). The ERN is known to be sensitive to various factors, including error probability (Ganushchak and Schiller, 2008) subject motivation, task salience, and the perceived cost of error (Lynn et al., 2010). For instance, Hajcak et al. (2005) and Rawls et al. (2020) demonstrated that high motivation conditions lead to increased amplitude and latency of the ERN compared to low motivation conditions, particularly for high-value trials. Additionally, both the ERN and the feedback-related negativity (FRN) show larger amplitudes with increasing reward size (Hajcak et al., 2005), irrespective of whether the reward is positive or negative (Gehring and Fencsik, 2001).

Several studies have reported larger ERN amplitudes when semantically similar stimuli were presented (Herrmann et al., 2004; Rawls et al., 2020), indicating the influence of stimulus characteristics and semantic salience on ErrP shape. In this study, we delved more deeply into this field and demonstrated that distinct stimuli exert a pronounced influence on the shape of ErrP. The observed significant effects of the stimulus set factor on the ERN in *No* responses suggest that the semantic characteristics of the visual stimuli may have an influence on this component. This influence may be attributed to several factors. First, Falkenstein et al. (2000) and Coles et al. (2001) proposed that the ERN is related to the processes of comparing two stimuli and their underlying representations. Our findings may indicate distinct processing of semantically diverse stimuli, meaning that for different feedback stimuli, outcome evaluation is associated with retrieving visually and semantically different images from memory. Secondly, prior research has emphasized the role of emotional content in stimulus-induced ErrPs (Wlotko and Federmeier, 2012), suggesting that emotionally salient stimuli could influence error processing and corresponding EEG responses. In contrast, the Friedman test revealed that the amplitude in *Yes* responses within the ERN latency was not affected by the *stimulus set* factor, probably because the neural processes of verification in *Yes* responses are less influenced by the stimulus content or its valence.

Following the ERN, we observed a positive peak with a latency of approximately 400–500 ms. We interpret this peak as the Pe, which is known to be larger for error events and associated with error awareness and subsequent behavioral correction (Endrass et al., 2007; Glazer et al., 2018; Pyasik et al., 2022). Typically, the Pe can be divided into early (frontally localized) and late (posterior) components, with the latter primarily associated with behavioral adjustments (Pyasik et al., 2022). Although we did not specifically focus on the separation of Pe components in our study, the fronto-central spatial distribution of the observed Pe suggests that the early Pe component predominated in our paradigm.

Our analysis demonstrated that the Pe component exhibited a distinct pattern in *No* responses. However, it did not show changes related to the *stimulus set* factor. In contrast, the positive components of the same latency in *Yes* responses demonstrated significant variability influenced by this factor. This suggests that ERN is more reactive to the visual content of erroneous stimuli, while Pe in *No* responses could reflect higher-level processes of integration, acceptance, and adaptation of error consequences (Ullsperger et al., 2014; Iwane et al., 2022).

Regarding the positive component with a latency range corresponding to Pe in *Yes* responses, we attribute this potential to activity similar to the feedback-related correct response positivity described by Cockburn and Holroyd (2018). These authors associate this potential with reward prediction error (RPE) systems, which are strongly related to the level of attention and salience of feedback stimuli (Walsh and Anderson, 2012). The observed effects of *stimulus set* factor on this late positive component in *Yes*-trials may be explained by differences in attractiveness of the different visual stimuli used. However, since our paradigm did not include explicit reward stimuli, we discuss this finding and its association with RPE with caution and acknowledge its speculative nature.

It is interesting that within the same experimental paradigm, some stimulus sets demonstrated the presence of both ERN and Pe (*objects*, *animals, emojis*), whereas in the *numbers* only Pe component was present. Our findings suggest that the presence of ERN is not a mandatory indicator of error awareness, aligning with a previous review (Xavier Fidêncio et al., 2022) reporting variable differences in the ERN and Pe across individuals. Some participants exhibited a virtually absent Pe while the ERN was prominent. Enriching the evidence for intersubject variability of Pe and ERN (Xavier Fidêncio et al., 2022), we described in this study that the frequency of error-related components in ERP curves can vary depending on the appearance of the stimuli used. It is important to emphasize, especially for future practical applications, that the observed differences in ErrPs across different stimulus sets make it challenging to effectively transfer classification algorithms for ErrP detection into a BCI loop: algorithms trained on a specific stimulus set may not generalize well to other stimulus sets.

Notably, we did not find ERN components within *No* responses for the *numbers*, which was unexpected given the clear distinctions between correct and incorrect stimuli within this set. Previous studies (Herrmann et al., 2004; Hajcak et al., 2005) have shown that the amplitude of ErrPs tends to increase as the error approaches correct performance. Therefore, we initially expected to find pronounced ErrPs for the *numbers*. This lack of pronounced disagreement reactions may be due to the short semantic distance between numbers, causing even erroneous numbers to be perceived as less distinct and thus not eliciting strong disagreement reactions.

Differences in the subjective attractiveness of stimuli across sets, influencing participant motivation, could explain the varied prominence of ErrP components. For example, participants may have found certain sets, such as the *objects*, more appealing and easier to visualize during the memorization interval than the *numbers*. However, since we did not conduct any surveys or assessments regarding the subjective attractiveness of the stimulus sets, we cannot provide direct evidence for this hypothesis.

It’s worth considering the impact of semantic connections among pictures within each stimulus set on the observed ErrPs. Specifically, errors in the *numbers* might be semantically closer to the correct answers compared to the erroneous stimuli in the *objects* and *animals*. Previous studies have reported a relationship between the amplitude of the ERN and the subjective distance between the error and the correct output (Hajcak et al., 2005; Hill, 2009; Spinelli et al., 2018; Iwane et al., 2022).

Our hypothesis posited that the shape of ErrPs could be influenced by the content of the stimuli, contributing to differences in ErrPs across the stimulus sets in our study. The objective of this study was to determine the extent to which the components associated with the object of attention modulate neural responses of agreement and disagreement. It was hypothesized that the ErrP waveform represents a mixture of picture-related visual potentials and *Yes*/*No*-related components.

To thoroughly investigate this hypothesis, we performed an additional analysis by subtracting the ERPs obtained during the memorization period from the *Yes-*/*No*-ERPs (Figure 5). The results show a high degree of similarity between the memorization ERPs and the *Yes-*/*No*-ERPs: during the ERN latency, both the memorization ERPs and the *No*-related ERPs show a negative deflection, although the *No*-related peak is more pronounced. A similar trend is observed for Pe, with the positive peak in this latency range also present in the ERPs recorded in memorization trials. At the same time, the *Yes*-ERPs differ significantly from the memorization-related responses. Permutation cluster-based tests indicated significant differences between *Yes-* and memorization-related ERPs within the time range where the ERN was described in *No* responses. On the other hand, *No*-ERPs did not show significant differences from memorization-ERPs. This suggests that the cortical responses in *No* reactions are highly modulated by responses to stimulus content. These findings align with the results of the Friedman test, which indicate that the amplitude of the ERN varies depending on the specific stimuli presented. The subtraction of memorization-ERPs from *Yes*-ERPs revealed a consistent pattern of negative and positive peaks in all stimulus sets. These peaks were most pronounced in the fronto-central area. The negative component in correct trials we found may be similar to previously described ERN-like activity, as identified by Coles et al. (2001) in correct trials. Coles et al. raised questions about the validity of theories specifically associating the ERN with error processing. The study proposed that output processing may be more strongly elicited by correct trials, potentially contaminating the ERN deflection in averaged ErrP. Our finding is also in line with the theory that the ERN-like activity primarily reflects a comparison between the representation of the correct answer and the actual output process, rather than solely being an indicator of error detection resulting from this process (Cruz et al., 2017). Given that this comparison process is also necessary for correct responses, it is plausible that the ERN is present in correct responses as well.

## 5 Conclusion

We want to highlight that previous studies have concentrated on error-related responses, whereas our findings indicate that there is also a need for further investigation into responses to correct inputs. The verification process is also conducted in correct trials. The results revealed that *Yes*-related potentials had components related to outcome processing that were invariant to stimulus content. This suggests that correct trials may provide insight into error processing cortical networks and could be highly valuable for practical application. Our results may be useful in the development of generalized classifications for ErrP detection in a zero-calibration approach across different paradigms (Cruz et al., 2018, 2022; Iwane et al., 2022), where the effects of stimulus scene on ErrPs should be taken into account.

## Data availability statement

The data supporting the conclusions of this article will be made available by the authors, without undue reservation.

## Ethics statement

The studies involving humans were approved by Institutional Ethics Committee of Immanuel Kant Baltic Federal University (protocol code № 40, 26.06.2023) for studies involving humans. The studies were conducted in accordance with the local legislation and institutional requirements. The participants provided their written informed consent to participate in this study.

## Author contributions

AB-A: Formal analysis, Investigation, Visualization, Writing – original draft. NiS: Data curation, Investigation, Methodology, Software, Visualization, Writing – original draft, Writing – review & editing. LY: Data curation, Investigation, Methodology, Writing – original draft, Writing – review & editing. AM: Formal analysis, Investigation, Writing – original draft. FG: Investigation, Writing – original draft. NaS: Funding acquisition, Project administration, Resources, Writing – original draft. AK: Conceptualization, Funding acquisition, Supervision, Writing – original draft, Writing – review & editing.
